# Colony-Stimulating Factor 3 Receptor Mutations and Variants in Hematological Malignancies

**DOI:** 10.3390/cancers17203378

**Published:** 2025-10-20

**Authors:** Clifford Liongue, Tarindhi Ratnayake, Alister C. Ward

**Affiliations:** 1 School of Medicine, Deakin University, Geelong, VIC 3216, Australia; c.liongue@deakin.edu.au (C.L.); s222416671@deakin.edu.au (T.R.); 2 IMPACT, School of Medicine, Deakin University, Geelong, VIC 3216, Australia

**Keywords:** aCML, AML, CMML, CNL, CSF3, CSF3R, G-CSF, G-CSFR, MDS, MPN

## Abstract

Colony-stimulating factor 3 (CSF3), additionally called granulocyte colony-stimulating factor (G-CSF), is the major cytokine regulating neutrophil production and also impacting their function. The actions of this cytokine are mediated through its unique receptor, the colony-stimulating factor 3 receptor (CSF3R). Several classes of pathogenic mutations in the *CSF3R* gene have been identified that have distinct biological properties and clinical impacts. This review provides an overview of CSF3R, the various pathogenic CSF3R mutations/variants and their biological effects. It also details the diseases to which they contribute, notably including chronic neutrophilic leukemia (CNL) and other myeloproliferative neoplasms (MPNs), myelodysplastic neoplasms (MDS), combined MDS/MPN disorders such as atypical chronic myeloid leukemia (aCML) and chronic myelomonocytic leukemia (CMML), as well as acute myeloid leukemia (AML) and lymphoid malignancies.

## Simple Summary

Cells can communicate via the release of small proteins that bind to specific receptors on the surface of other cells, with so-called ‘cytokine receptors’ particularly important for blood and immune cells. One such cytokine receptor, named ‘colony-stimulating factor 3 receptor’ (or ‘CSF3R’), regulates the production and function of a group of white blood cells called neutrophils. Mutations and variants in the gene that produces CSF3R have been identified in blood and immune cell cancers. Here, we review the different CSF3R forms and their role in specific diseases.

## 1. Introduction

Colony-stimulating factor 3 (CSF3), also commonly referred to as granulocyte colony-stimulating factor (G-CSF), is a cytokine that plays a key role in the generation and function of neutrophils [1], critical cells in inflammation and innate immunity particularly against bacterial and fungal pathogens [2]. The biological actions of CSF3 are mediated following binding to a specific receptor, CSF3R (also termed G-CSFR), expressed on the surface of neutrophils and their precursors [3].

Mutations and germline variants in the *CSF3R* gene have been identified that fall into a number of classes with distinct impacts both biologically and clinically. These are differentially implicated in a variety of hematological malignancies, including myeloproliferative neoplasms (MPNs), particularly chronic neutrophilic leukemia (CNL), but also extending to myelodysplastic neoplasms (MDS) and various other myeloid diseases such as atypical chronic myeloid leukemia (aCML), chronic myelomonocytic leukemia (CMML) and acute myeloid leukemia (AML), as well as lymphoid and other malignancies [4,5,6,7,8,9]. This review describes the structure and function of CSF3 and details the various classes of CSF3R mutations/variants and how they impact signaling. It then discusses the hematological malignancies with which they are associated along with the collaborative genes that contribute to disease and therapeutic approaches being applied to combat these diseases.

## 2. CSF3R Structure and Function

**Figure 1:**
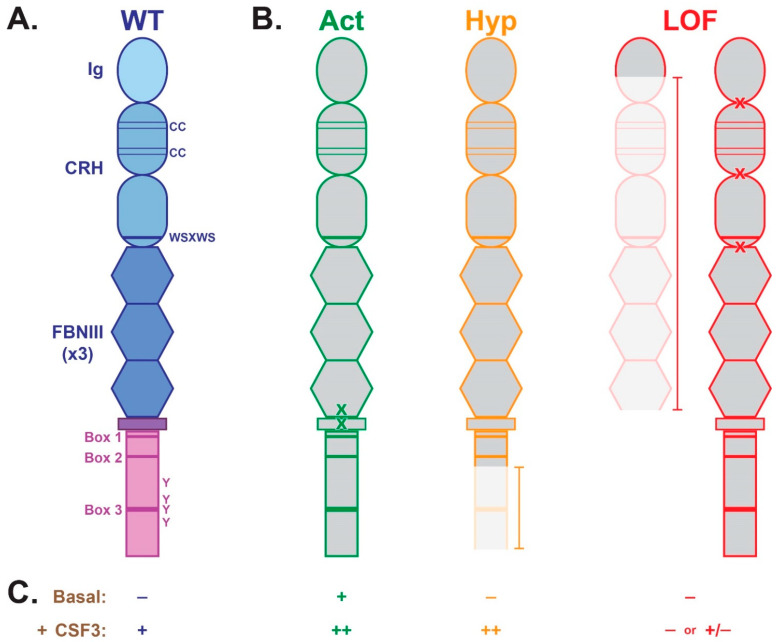
Structure and function of the CSF3R: normal and pathogenic mutations/variants. (**A**) Schematic representation of the wild-type (WT) CSF3R, showing the immunoglobulin (Ig)-like domain, cytokine receptor homology (CRH) domain and triplicate fibronectin type 3 (FNIII) domains that collectively constitute the extracellular region (blue), the transmembrane region (purple), and the intracellular region (pink), with WSXWS and Box motifs along with important cysteine pairs (CC) and tyrosine (Y) residues indicated. (**B**) The major classes of pathogenic *CSF3R* mutations/variants—activating (Act, green), hyperactive (Hyp, orange) and loss-of-function (LOF, red)—together with the location of respective common enabling (green crosses) and disabling (red crosses) point mutations and deletions (orange and red lines). (**C**) Relative signaling activity of the various CSF3R receptor forms basally and following CSF3 stimulation (+CSF3).

The CSF3R is a transmembrane protein expressed almost exclusively by cells within the neutrophil lineage and their precursors [3]. The protein has a substantial extracellular region, comprising an N-terminal immunoglobulin (Ig)-like domain, a cytokine receptor homology (CRH) domain, with two conserved cysteine pairs and a WSXWS motif, and three fibronectin type 3 (FNIII) domains. A short transmembrane (TM) region connects this to an intracellular region, containing Boxes 1–3 as well as tyrosine and other sequence motifs required for its signaling function (Figure 1A) [10]. The CSF3R is inactive basally, but becomes transiently activated in the presence of CSF3 (Figure 1C).

**Figure 2:**
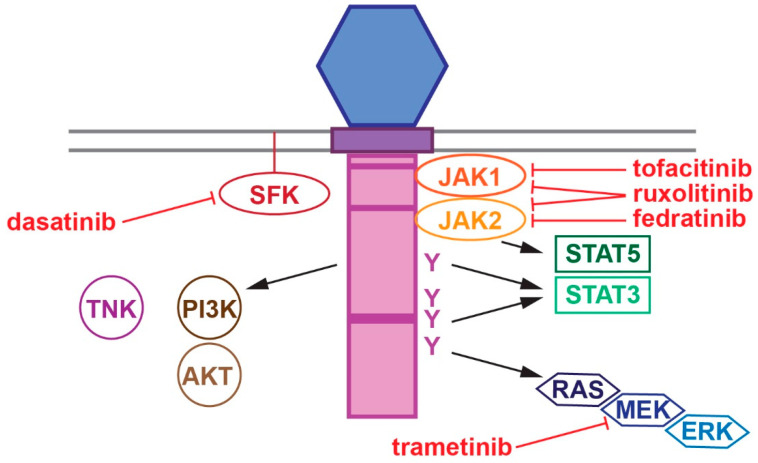
Intracellular signaling via the CSF3R and its therapeutic targets. Schematic representation of the transmembrane and intracellular regions of the CSF3R, showing some of the key intracellular pathways activated and relevant therapeutic agents targeting them.

CSF binds to the extracellular CRH region of the CSF3R leading to structural alterations that facilitate activation of the receptor-associated Janus kinases (JAKs), membrane-bound SRC-family kinases (SFKs) and other tyrosine kinases such as TNK [11,12,13,14]. This enables phosphorylation of the four intracellular tyrosine residues of the CSF3R, generating docking sites for many signaling molecules, such as signal transducer and activator of transcription (STAT) proteins, particular STAT3 and STAT5 [15,16], as well as upstream activators of the RAS/MEK/ERK and PI3K/AKT pathways [13,17,18] (Figure 2). Collectively, these impact neutrophilic lineage commitment, differentiation, proliferation, survival, migration and effector functions [19]. This conserved signaling system contributes to basal neutrophil production throughout the life-course [20,21], but is especially important in ‘emergency’ settings, such as acute infection where CSF3 expression is markedly increased [22].

## 3. CSF3R Mutations and Variants Associated with Hematological Malignancy

An increasing number of *CSF3R* gene mutations and variants have been identified in hematological malignancies and other disorders. These are most commonly somatically acquired mutations that act in a dominant manner, although there are also important germline variants and/or recessive forms. These mutations/variants can be grouped into distinct classes based on their position and biological impact (Figure 1B,C). The mechanistic aspects have been principally investigated by the transduction of constructs expressing the various CSF3R forms into hematopoietic cells lines or bone marrow cells in concert with primary patient cells. Animal models generated by transplantation of transduced bone marrow or gene knock-in approaches have underpinned preclinical studies.

### 3.1. Activating

This class of mutation/variant normally involves residues closely adjacent to or within the CSF3R transmembrane domain, with the most common being T618I followed by T640N [6,23,24,25] (Figure 1B). They typically represent acquired mutations [6,7]—although there exist important germline variants [24]—and act in a dominant manner [6,24]. Collectively, these mutations/variants function by stabilization of helix–helix interactions around the membrane, with loss of glycosylation contributing for at least some mutants [23,24,26,27]. This causes constitutive activation of the CSF3R with robust signaling in the absence of CSF3 that is further enhanced by CSF3 stimulation [24,28] (Figure 1C). There is, however, selectivity in the pathways activated, which include JAK2 via STAT3 and STAT5, SFKs via TNK, AKT and ERK [6,7,24], with enhanced ROS production also observed [7]. These mutations have been demonstrated to facilitate CSF3-independent proliferation, survival and/or differentiation of cell lines [6,7,23], transduced bone marrow [29], or patient cells [23,30]. Retroviral transduction of bone marrow cells with several variants caused neutrophilic hyperplasia when transplanted into irradiated mice [24,28,31]. Alternative activating forms have been described: the somatic W341C mutant in the extracellular region, in which disulphide-mediated receptor dimerization mediates to constitutive activation [32,33], and the germline P733T variant in the intracellular region that activates CSF3R signaling by a yet unknown mechanism [34].

### 3.2. Hyperactive

These CSF3R forms are typically the result of acquired mutations that truncate the intracellular region [35]. The majority represent nonsense mutations that can occur across a wide span of this domain, such as Q741* and W791*, but other mutations can cause frameshifts and other genetic aberrations that cause premature termination of the native coding sequence [36,37,38] (Figure 1B). Collectively, hyperactive CSF3Rs principally act in a dominant manner to ablate normal control mechanisms. These include impaired internalization/trafficking, due to the loss of a conserved di-leucine containing motif in Box 3 [39,40] and another downstream motif [41], in concert with the absence of recruitment sites for various negative regulators, including SHP-1 [42], WSB2 [43], SOCS3 [41], CISH and SHIP [44], depending on the exact site of the truncation. Hyperactive CSF3R forms are inactive basally, but show enhanced signaling (length and magnitude) following CSF3 stimulation (Figure 1C), due to a significantly decreased “off-rate” [16,39,45], and hypersensitivity to ligand [4,40]. Of particular importance are the impacts on STAT5 [39,40,42,46], AKT and other pathways downstream of PI3K [47,48] and SFKs [6]. Hyperactive receptors have also been shown to increase ROS production [49] and induce a pro-inflammatory response [50], potentially creating a mutagenic environment important for transformation. Animal models expressing hyperactive CSF3Rs displayed hyper-responsiveness to CSF3 and/or elevated neutrophil numbers [51,52,53], while other studies demonstrated that exogenous CSF3 mediated a strong clonal HSC advantage for hyperactive CSF3R mutations [54]. However, unlike activating mutations, hyperactive CSF3R forms were insufficient to lead to transformation [31,55], but enhanced leukemogenesis in concert with other genetic mutations [56] or compounded with an activating mutation [55]. In addition to truncations, more subtle sequence changes have also been identified, including a germline P784T variant able to inhibit internalization, leading to CSF3 hypersensitivity [8] and an acquired N653K_R654del mutation that mediates prolonged signaling following CSF3R stimulation [57].

### 3.3. Loss-of-Function

Another class of mutation/variant typically impacts the sequences encoding the CSF3R extracellular domain. They often represent large truncations of the CSF3R, a missing part of the extracellular region and all of the transmembrane and intracellular regions, including the germline Y56fs99* [58], S322Gfs*29 [59], S624Rfs*177 [60] and W547* [8] variants as well as the acquired S319Gfs*29 mutation [61]. Other germline variants impact key structural residues within the CRH domain, notably including a conserved di-proline “hinge” motif, P229H [62] and P230L [63], but also A119T in this domain [8] (Figure 1B). Collectively, these CSF3R forms exhibit a dominant loss-of-function (LOF) phenotype, with signaling abolished or significantly ablated even in the presence of CSF3 (Figure 1C), and severe impacts on proliferation and differentiation [8,21,62]. Alternative CSF3R forms that may be included in this class are the germline intracellular variants, E808K that retains some function, but can act in a dominant manner to reduce colony formation [5], the partial LOF P785T [8] and an acquired mutation adjacent to a cryptic splice-donor site that resulted in elevated levels of an alternatively spliced transcript encoding a P708Afs*34 form of CSF3R unable to transduce signals for proliferation or differentiation [64].

## 4. Hematological Malignancies Associated with CSF3R Mutations/Variants

A range of hematological and other diseases, particularly those affecting the myeloid lineage, have been associated with both acquired mutations and germline variants of CSF3R (Table 1), with specificity between the different CSF3R forms and particular diseases.

### 4.1. Myeloproliferative Neoplasms (MPNs)

MPNs represent a collection of disorders in which a single myeloid lineage is expanded [85], including chronic neutrophilic leukemia (CNL), a relatively rare MPN with characteristic neutrophilia typically associated with splenomegaly and a generally poor prognosis [86]. Acquired activating CSF3R mutations have been identified in around 90% of CNL patients and represent the most predominant genetic lesion for this cancer type [87,88], such that these mutations serve as a diagnostic criteria for the disease [85]. The majority are T618I mutations, but there are many others [6,87,89], although the T618I mutation has been associated with more adverse clinical characteristics [86]. Such activating CSF3R mutations have also been observed in CNL secondary to MDS [90] or CML [91]. A substantial subset of CNL patients with activating CSF3R mutations also contain a hyperactive mutation on the same allele [87,92]. A familial form of CNL is also caused by a germline T618I variant [83], although none in a large family cohort transformed to AML [30]. Autosomal dominant hereditary neutrophilia, in which neutrophils are also chronically elevated with the potential for splenomegaly, is mediated by the alternative activating germline variant T640N that exhibits complete penetrance [24,93,94]. In contrast, a patient with MPN-NOS likely secondary to so-called ‘CBL-syndrome’ was found to harbor a germline Y56Sfs99* mutation, presumed to be LOF, suggesting an alternative etiology in this case [58].

### 4.2. Myelodysplastic Neoplasms (MDS)

MDS encompasses a group of disorders characterized by clonal hematopoiesis, with morphological dysplasia, persistent cytopenia(s) and a propensity to progress to AML or bone marrow failure [85]. A different spectrum of CSF3R mutations have been implicated in MDS, including some of the less common mutations/variants. Thus, the germline LOF variant E808K has been shown to confer susceptibility to high risk MDS [5] with the germline W547* also implicated in MDS susceptibility [8], while acquisition of the hyperactive N653K_R654del mutation has been identified in a patient with MDS [57]. In MDS secondary to CN, various acquired hyperactive CSF3R mutations occur at high frequency [81].

### 4.3. MDS/MPN

Disorders in the MDS/MPN category, with features of both myelodysplasia and myeloproliferation [85], have also been associated with a mix of CSF3R mutation types. In atypical chronic myeloid leukemia (aCML), characterized by dysplastic neutrophils and circulating precursors, CSF3R mutations have been found in ~18% of cases [6,9,72,73]. Once again activating mutations, including those compounded with hyperactive mutations, have been identified most commonly, but also other mutations including the CSF3R-SPTAN1 translocation [6,9,38,73,95]. The LOF E808K germline variant has also been observed in aCML [96] including in a patient that developed the disease following MDS [28]. In chronic myelomonocytic leukemia (CMML), CSF3R mutations occur at an even lower frequency of <2%, with these associated with poorer outcomes [74,87]. Acquired activating including with compound hyperactive CSF3R mutations are again seen most frequently [7,9,74,75,97,98,99,100], while the germline P733T variant is associated with predisposition to this disease [34]. Acquired activating CSF3R mutations have also been observed in MDS/MPN-U [99,101].

### 4.4. Acute Myeloid Leukemia (AML)

AMLs are rapidly progressing myeloid neoplasms, with characteristic clonal expansion of myeloid precursors in the bone marrow causing ineffective hematopoiesis and bone marrow failure [85]. A variety of CSF3R mutations have been collectively associated with ~2% of AML cases across various cohorts [6,7,25,33,73,80,102]. Collectively these CSF3R mutations are associated with adverse prognosis [84] and increased risk of relapse [79] in AML. In the case of de novo AML, activating mutations such as T618I predominate, but significant numbers of hyperactive and other mutations have also been identified [6,7,64,73,76,78,103]. A similar CSF3R mutation spectrum has been detected in relapsed and secondary AML at even higher rates [78]. An acquired activating T618I mutation was also observed in AML secondary to chronic eosinophilic leukemia [104]. By contrast, in AML secondary to congenital neutropenia, hyperactive mutations take precedent—indeed they are the most common mutations found in this patient cohort at nearly 90% [81,82]. The germline activating P733T variant also mediates susceptibility to AML [84].

### 4.5. Lymphoid and Malignancies

While CSF3R mutations/variants are mainly associated with myeloid diseases, they have also been identified particularly in B-cell acute lymphoblastic leukemia (B-ALL) and multiple myeloma (MM), most likely a consequence of CSF3R expression in early hematopoietic progenitors. Once again, acquired activating and hyperactive CSF3R mutations have been identified [78,103,105]. Amongst the germline variants, the activating P733T form has been associated with susceptibility to B-ALL to an even greater extent than it does for AML [84], while the partial LOF A119T and P784T have been identified in B-ALL and MM [8]. Interestingly, both activating variants and the LOF A119T have been associated with MM susceptibility [8,95].

## 5. Co-Operating Gene Mutations

Neoplasia is a multi-step process requiring multiple genetic and other changes. A number of studies have provided insights into the gene mutations that co-operate with CSF3R mutations in this manner. What has emerged is both overlap and specificity in the co-operating gene mutations between the different CSF3R mutation/variant classes and the various disease types in which they are found (Table 1).

Importantly, additional CSF3R mutations have been observed. These are most common in CNL in the form of compound activating and hyperactive mutations in around 21% of cases [87,92], including cases where one mutation is germline [106]. This situation has also been observed slightly less frequently in aCML and CMML [99,107], with the clinical impact still unclear. However, in other settings, additional CSF3R mutations have been associated with progression to more severe disease. For example, transformation of CN to AML has been shown to correlate with additional acquired activating [29] or hyperactive [108] mutations. Furthermore, a patient who developed an MDS-like disorder secondary to hereditary neutrophilia acquired another activating mutation (T617N) along with their germline activating T640N variant [24], while in AML secondary to high-risk MDS, blast cells had become homozygous for the E808K variant [109].

In CNL, the most common co-operating mutations identified are in the epigenetic regulators ASXL1 and SETBP1, with EZH2 also in this category [73,87]. The SETBP1 mutants have been demonstrated to promote self-renewal of CSF3R-mutated progenitors and block terminal differentiation due to activation of a Myc program leading to accelerated leukemic development in a mouse model [89]. Concurrent SETBP1 mutations have been associated with evolution to CMML [68]. In a case of CNL secondary to MDS the patient acquired a SETBP1 mutation first and then a CSF3R [90]. Murine studies have shown that combined CSF3R and ASXL1 mutations lead to expansion of myeloid biased hematopoietic precursors and neutrophilia, with ASXL1 mutations enhancing mutant CSF3R-mediated differentiation as well as increasing inflammatory pathways [110]. Concurrent ASXL1 mutations have been demonstrated to lead to a poorer prognosis, including evolution into AML [68], while a case that progressed to MPAL involved sequential ASXL1 and RUNX1 mutations [106]. Other common gene mutations include splicing factors, such as SRSF2 (and U2AF1), and lower frequency of mutations in transcription factors, like TET2 and GATA2, and other signaling proteins, such as CBL and NRAS, [34,73,86,87,111]. A concurrent CBL mutation was also observed in a case of MPN-NOS [58].

In aCML, activating CSF3R mutations were also commonly found in association with ASXL1 and SETBP1 mutations, along with SRSF2 and TET2, but also EZH2 and CEBPA [9,73]. In CMML, CSF3R T618I mutations were enriched in ASXL1 mutations but exclusive of TET2 mutations, with the ASXL1 mutated group showing more adverse outcomes, whereas non-T618I mutations harbored a mix of ASXL1 and TET2 mutations, with SRSF2 also commonly mutated [75,98]. A case of CMML with the germline LOF E808K variant possessed TET2 and SRSF2 mutations as well [96]. Cooperation has also been observed with mutations in other key signaling components, including KRAS and RUNX1 in CMML [99]. A MDS/MPN-U patient possessed concurrent TET2, SETBP1 and PTPN11 mutations [99].

In AML, the majority of CSF3R mutations are associated with mutations in specific transcription factors, either one of the constituent sub-units of core-binding factor (CBF)—RUNX1 or CBFB—or CEBPA [76,77,78,80,112]. Activating CSF3R mutations, acquired or germline, have been shown to co-operate with both CBF and CEBPA mutations, but hyperactive mutations only co-operate with the CBF ones [78,80,84]. Subsequent functional studies have demonstrated that the transcription factor mutations typically precede the activating CSF3R mutation, as shown for both CEBPA [113] and RUNX1:RUNXT1 t(8;21) [114]. CEBPA has previously been shown to be essential for neutrophil differentiation downstream of CSF3R [115], with CEBPA mutations demonstrated to block the ability of CSF3R mutations to activate differentiation but not proliferation in mice [113]. Concurrent RUNX1 mutations have also been shown to accelerate leukemogenesis, with increased proliferation and impaired differentiation, probably due to the suppression of CEBPA expression [114,116]. RUNX1-RUNX1T1 in concert with activating CSF3R mutations provided a clonal advantage, with increased self-renewal and blast-like morphology [117]. CSF3R mutations have been associated with poorer outcomes in AML patients with concurrent CEBPA, but not CBF mutations [80,118]. Other genes which showed a significant co-occurrence with CSF3R mutations in AML are signaling proteins, such as, KIT, FLT3 and also NRAS with both activating and hyperactive CSF3R mutations [78,80,119], as well as NPM1 [78], especially in concert with CEBPA [80], and the transcription factor IKZF1 [120]. In AML secondary to CN, RUNX1 was found to be mutated in the majority of cases [81]. Introduction of concurrent CSF3R and RUNX1 mutations in CN-driven iPSCs resulted in hyperproliferation in response to G-CSF [121], while studies in an ex vivo mouse model showed decreased differentiation and increased clonicity in concert with increased pro-inflammatory signals [122]. ASXL1 mutations also show high prevalence [106], but not those in signaling proteins [119].

## 6. Therapeutic Considerations

Elucidation of the important causative role for CSF3R mutations and critical downstream signaling pathways in the etiology of disease has underpinned multiple approaches to therapy (Figure 2). This has revealed intrinsic differences between different CSF3R forms. Thus, activating CSF3R mutants have been found to preferentially activate JAKs and be sensitive to the JAK1/2 inhibitor ruxolinitib [6,7,28], but not to the SFK inhibitor desatinib that targets SFKs [6,7]. For example, CSF3R T618I mutant patient cells showed an IC50 of 127 nM for ruxolinitib and >1000 nM for desatinib [6]. In contrast, hyperactive CSF3R mutants favor SFK activation and were sensitive to desatinib but resistant to ruxolinitib in the absence of CSF3 [6]. Another study in transduced cell lines showed differential sensitivity of wild-type CSF3R, activating, hyperactive and compound mutants to ruxolitinib (respective IC50s of 154 nM, 118 nM, 31 nM and 427 nM without CSF3 and 374 nM, 273 nM, 83 nM and 660 nM with CSF3), with the compound mutant most resistant also to dasatinib [55]. However, ruxolitinib was shown to be effective in mice transplanted with bone marrow transduced with the activating T618I mutant, with reduced WBC and spleen weight, but increased body weight [31] and in patients [6]. Subsequent phase II clinical trials have investigated the use of JAK inhibitors in relevant clinical contexts. The first of these examined ruxolitinib in CNL and aCML patients (NCT02092324) and showed a significantly enhanced response rate in patients with mutant compared to wild-type CSF3R (54% vs. 9%; *n* = 25) and improvements in WBC parameters and spleen size (*n* = 29) [70]. The other trial investigated fedratinib in CNL, aCML and MDS/MPN patients (NCT05177211), with responders again enriched in the mutant CSF3R cohort (83% vs. 42%; *n* = 24). Favorable responses to ruxolitinib have also been observed in multiple case studies, including a patient with a compound mutation [123] and another with T618I CNL and renal abscesses, where treatment improved both the CNL and the infection, the latter attributed to its anti-inflammatory properties [124]. Alternatively, a patient with B-ALL carrying a hyperactive CSF3R mutation responded favorably to dasatinib in combination with chemotherapy [105], suggesting this treatment may also have clinical application. Other studies in preclinical mouse models have identified enhanced MAPK signaling to be essential for leukemogenesis induced by CSF3R activating and compound mutants [55,125], with the MEK inhibitor trametinib found to inhibit leukemic transformation, being both cytostatic as well as selectively ablating the mutant clone [55]. Another study showed that the enhanced MAPK signaling resulted in dependence on dual specificity phosphatase 1 (DUSP1) that augmented survival signaling, and further that combined use of a DUSP1 inhibitor in concert with trametinib eradicated leukemia [126]. Alternatively, Aurora kinase inhibitors have been demonstrated to reduce proliferation, viability and clonogenicity in a CSF3R T618I cell line, similar to their effects in BCR-ABL1-mediated CML and JAK2V617-mediated MPN providing an alternative therapeutic avenue to pursue [127].

Important new insights are also emerging regarding how the wider genomic context of CSF3R mutations are important in therapeutic choice. For example, the combination of activated CSF3R and RUNX1 mutation has been shown to result in upregulation of the Hedgehog pathway, including GLI2, and subsequent sensitivity to the GLI inhibitor GANT61 in patient hematopoietic cells [117], as well as upregulation of BAALC leading to MK2a phosphorylation in patient-derived iPSCs that was sensitive to the MK2a inhibitor CMPD1 [128]. Furthermore, patients with concurrent CSF3R and NRAS mutations were responsive to combined ruxolitinib and trametinib therapy [69]. Finally, concurrent CSF3R and SETPBP1 mutations have shown sensitivity to LSD1 that targets the upregulated Myc induced by mutant SETBP1 [89]. 

## 7. Additional Oncogenic Roles for CSF3R

In addition to the important role played by *CSF3R* mutations in hematological cancers, CSF3R signaling contributes to oncogenesis by other mechanisms including when the CSF3R gene is not mutated. For example, in hematological cancers SRSF2 mutations have been shown to increase levels of a normally minor CSF3R transcript encoding a truncated V684Afs*34 CSF3R isoform shown to inhibit differentiation [129]. In addition, the RUNX1-RUNX1T1 fusion protein has been found to induce CSF3R expression levels, resulting in enhanced proliferative responses [130]. Meanwhile, CSF3R has been shown to be expressed in a range of solid tumor samples, with higher expression generally associated with a poorer prognosis, such as in glioma [131]. This can be due to direct effects on the tumor cell, promoting adhesion and invasion in bladder cancer [132], migration and survival in ovarian cancer [133], proliferation and migration in gastric and colon cancer [134], migration in breast cancer [135], and proliferation particularly of the cancer stem cell population in neuroblastoma [136] and glioma [131]. However, it can also exert a pro-tumorigenic effect through its impact on tumor-associated immune cells [137]. For example, on pro-tumorigenic macrophages in breast cancer [135], glioma [131], colon and pancreatic cancer [138], as well as T cell subsets in colon cancer [139], and also in angiogenesis in the context of Ewing sarcoma [140]. This is particularly relevant given the use of CSF3 to restore neutrophil numbers following chemotherapy for various solid tumors [141]. A smattering of *CSF3R* mutations have also been reported in on-line databases for solid tumors, including bladder cancer and head and neck squamous cell carcinoma, albeit at very low frequencies.

## 8. Conclusions

Signaling by CSF3 through the CSF3R contributes in an important manner to neutrophil homeostasis, but especially in the context of inflammation, infection and their respective resolution [1]. The effectiveness, specificity and transient impact of CSF3 have underpinned its wide usage in clinical settings in which neutrophil numbers are depleted [142]. However, disruption of this exquisite system by CSF3R mutations can result in hematological malignancies and other diseases. Mutations that no longer require or are hyperresponsive to CSF3 result in the overproduction of neutrophils and their precursors with additional cooperating mutations leading to neoplasia. Intriguingly, other mutations that block CSF3 action also contribute to such disorders, most likely acting by blocking differentiation. Understanding the molecular mechanisms underpinning the disruptions to normal function is ushering in a wave of therapies that aim to mitigate the effects of these mutations and rebalance the system.

## Figures and Tables

**Table 1:** Hematological disorders associated with CSF3R mutations/variants including concurrent mutated genes.

Diseases	Genetics	Frequency	Major Mutation Type(s)	Common Concurrent Mutations	References
CNL	Somatic, dominant	76.0%	Act (T618I) 91% ⟶ with Hyp (various) 21%	ASXL1 82%, SETBP1 67%, SRSF2 30%, EZH2 20%	[6,9,34,65,66,67,68,69,70,71,72,73]
aCML	Somatic, dominant	18.5%	Act (T618I) 89% ⟶ with Hyp (various) 20%	ASXL1 65%, SETBP1 41%, SRSF2 41%, TET2 30%	[6,9,65,66,70,72,73]
CMML	Somatic, dominant	1.6%	Act (T618I, P733T) 48% ⟶ with Hyp (various) 10%	ASXL1 85%, TET2 43%, SRSF2 33%	[9,34,66,69,74,75,76]
AML	Somatic, dominant	1.7%	Act (T618*) 74%, Hyp (various) 25%	CBF 43%, CEBPA 35%, KIT 19%, FLT3 17%	[7,9,23,25,28,76,77,78,79,80]
AML: 2° to CN	Somatic, dominant	88.9%	Hyp (various) 100%	RUNX1 67%	[81,82]
Hereditary neutrophilia	Germline, dominant	100% penetrant	Act (T640N) 100%	??	[24]
Familial CNL	Germline, dominant	100% penetrant	Act (T618I) 100%	??	[83]
Susceptibility to high-risk MDS	Germline, dominant	9.7% [OR = 12.5]	LOF (E808K)	??	[5]
Susceptibility to AML/ALL	Germline, dominant	1.9–7.8% [OR = 1.5 → 5]	Act (P733T)	??	[84]

??: unknown.

## Data Availability

No new data were created or analyzed in this study. Data sharing is not applicable to this article.
